# Adsorption of iodine in metal–organic framework materials

**DOI:** 10.1039/d0cs01192d

**Published:** 2022-04-01

**Authors:** Xinran Zhang, John Maddock, Tina M. Nenoff, Melissa A. Denecke, Sihai Yang, Martin Schröder

**Affiliations:** School of Chemistry, University of Manchester Manchester M13 9PL UK sihai.yang@manchester.ac.uk m.schroder@manchester.ac.uk; Materials, Physics and Chemical Sciences Center, Sandia National Laboratories Albuquerque NM 87185 USA; Division of Physical and Chemical Science, Department of Nuclear Applications, International Atomic Energy Agency, Vienna International Centre PO Box 100 1400 Vienna Austria

## Abstract

Nuclear power will continue to provide energy for the foreseeable future, but it can pose significant challenges in terms of the disposal of waste and potential release of untreated radioactive substances. Iodine is a volatile product from uranium fission and is particularly problematic due to its solubility. Different isotopes of iodine present different issues for people and the environment. ^129^I has an extremely long half-life of 1.57 × 10^7^ years and poses a long-term environmental risk due to bioaccumulation. In contrast, ^131^I has a shorter half-life of 8.02 days and poses a significant risk to human health. There is, therefore, an urgent need to develop secure, efficient and economic stores to capture and sequester ionic and neutral iodine residues. Metal–organic framework (MOF) materials are a new generation of solid sorbents that have wide potential applicability for gas adsorption and substrate binding, and recently there is emerging research on their use for the selective adsorptive removal of iodine. Herein, we review the state-of-the-art performance of MOFs for iodine adsorption and their host–guest chemistry. Various aspects are discussed, including establishing structure–property relationships between the functionality of the MOF host and iodine binding. The techniques and methodologies used for the characterisation of iodine adsorption and of iodine-loaded MOFs are also discussed together with strategies for designing new MOFs that show improved performance for iodine adsorption.

## Abstract 2

The chemistry and applications of metal–organic framework materials for iodine and polyiodide capture and storage are reviewed.

## Graphical abstract

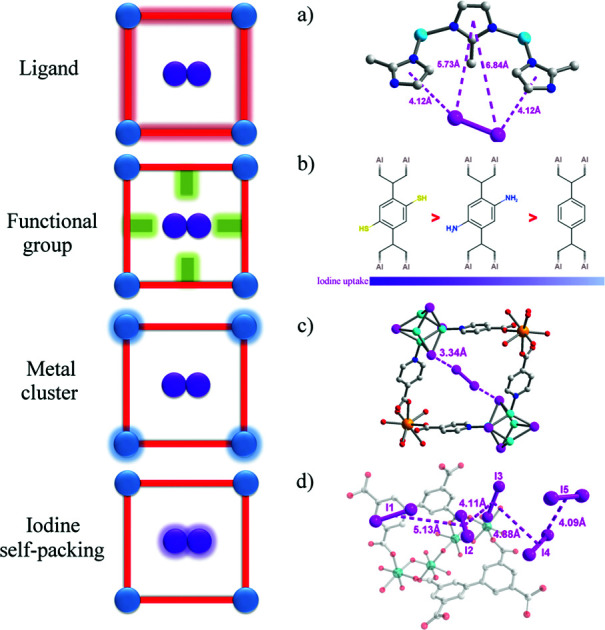

## Introduction

**Fig. 1:**
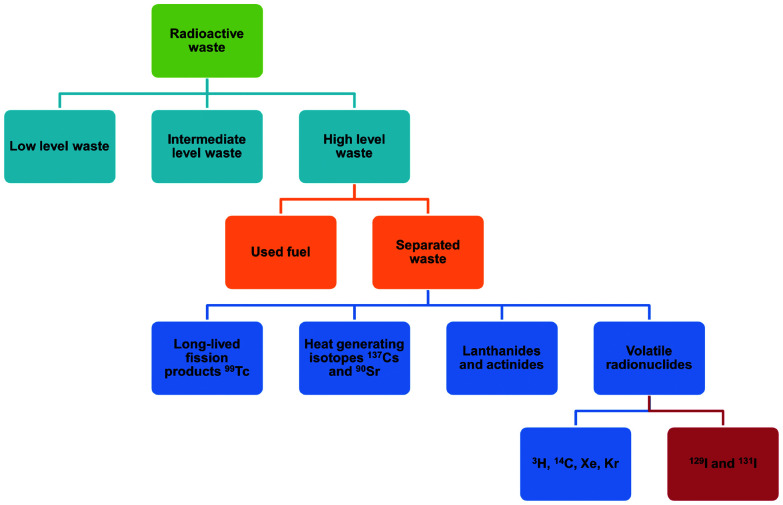
Classification of nuclear wastes.

**Fig. 2:**
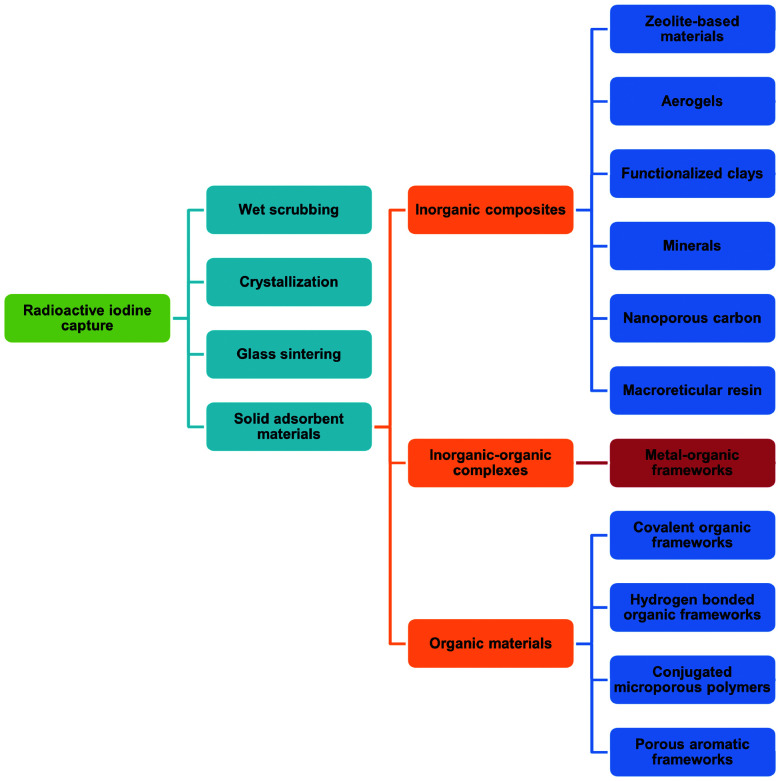
State-of-the-art methods for removal of radioactive iodine.

Uranium used in nuclear fission has an energy density that is seven orders of magnitude higher than that of coal or gasoline and accounts for 10% of the world's total electricity generation in 2020 from 440 reactors.^1^ This is projected to increase over the coming decade. Nuclear energy is a relatively clean energy source at the point of use compared to fossil fuels, which produce enormous volumes of CO_2_, SO_*x*_, NO_*x*_ and particulate matter.^2^ However, management of radioactive waste is a challenging issue as inappropriate handling or disposal can lead to the unintended release of radionuclides, thus posing significant risks to health and of long-term contamination of the environment. It is particularly challenging to deal with radionuclides that are volatile (*e.g.*, ^3^H, ^85^Kr, ^129^I) and which can readily spread through the atmosphere or *via* solution in water (Fig. 1). The key isotopes of concern are ^129^I and ^131^I, which have a half-lives of 1.57 × 10^7^ years and 8.02 days, respectively.^3^ Although with a much shorter half-life, the latter can directly interfere with human metabolic processes, causing serious health problems.^4^ Solid materials with open structures such as zeolites,^5^ chalcogels,^6^ microporous polymers^7^ and covalent–organic framework^8^ materials have also been investigated for iodine capture due to their high adsorption capacity and promising reusability.^9^ However, these materials (apart from zeolites) generally lack long-range structural orders and have random adsorption sites, precluding the study of host–guest interactions and thus hindering understanding of the mechanism of action and the design of improved materials. A general introduction on iodine adsorption in these materials is given below. Once iodine is captured, sequestration is a key target for the prevention of emission back into, and removal from, the environment. Recently developed techniques to achieve iodine sequestration include wet scrubbing,^10^ mineral crystallisation^11^ and glass sintering (Fig. 2).^12^

Metal–organic frameworks (MOFs) are a class of crystalline porous materials that possess high surface areas, tuneable structures and can have high chemical stability.^13^ The majority of current research is focused on the use of MOFs for gas adsorption and storage.^14^ However, they also exhibit potential for toxic waste elimination, including removal of corrosive gases,^15^ separation of noble gases^16^ and adsorption of heavy metals.^17^ Recently, MOFs have been shown to adsorb a wide range of radionuclides,^18,19^ including barium,^20^ uranium,^21^ thorium,^22^ iodine and triiodide (I_3_^−^).^23^ More recently, novel MOF materials have also been synthesised based on actinides metal clusters.^24,25^ The application of MOFs for iodine adsorption has not been systematically reviewed previously, and herein, we discuss the latest progress on the adsorption and binding of iodine in robust MOF materials. The crystalline nature of MOFs enables investigations of the host–guest binding interactions for iodine-loaded materials at crystallographic and molecular resolution. The dynamics of iodine adsorption in MOFs are also discussed to afford insights for the design of future systems with improved properties. Furthermore, we explore the potential utilisation of iodine-loaded MOFs, for example in heterogeneous catalysis.

## Iodine adsorption in solid sorbents

### Zeolites

Zeolites are a class of microporous inorganic materials consisting of tetrahedral MO_4_ moieties (*e.g.*, M = Si, Al) that are connected *via* corner-sharing oxygen centres to form intersected voids.^25^ The channels in zeolites typically have narrow windows (size < 10 Å) that provide an ideal environment for substrate binding and activation. Various types of zeolites with diverse framework topologies, such as ZSM-5 (MFI type),^26^ NaX and NaY zeolites (FAU type)^27^ have been tested for iodine adsorption with silver-containing zeolite mordenite (MOR type) being the most widely used for the capture of radioactive iodine.^28,29^ This approach is based upon promotion of formation of AgI upon inclusion of iodine which greatly simplifies the reprocessing of the zeolite.

Typical MOR-type zeolitic frameworks include M_*x*_Al_2_Si_10_O_24_**·**7H_2_O (M = Ca, *x* = 1; M = Na, K, *x* = 2) that comprise of 12-membered rings with a pore diameter of 7.0 × 6.5 Å and 8-membered rings incorporating windows of 5.7 × 2.6 Å. These features facilitate the diffusion of iodine vapour through the structure. Ion-exchange reactions afford zeolites with high Ag(i) ion loading, and these sorbents exhibit excellent stability when exposed to a stream of iodine. The mechanism of host–guest binding has been investigated by pair distribution function (PDF) analysis,^30^ and two different forms of AgI can be generated by reaction of iodine with these Ag-loaded zeolites: α-AgI is retained within the pores, while larger nanoparticles of γ-AgI reside on the surface of the zeolite. Recently, a novel hydrophobic all-silica zeolite HISL (hydrophobicity intensified silicate) has been developed for iodine adsorption. Its narrow channels (5.5 × 5.1 Å) are advantageous for selectively trapping iodine, the kinetic diameter of which is 4 Å, and an uptake of 0.53 g g^−1^ has been obtained for HISL under humid conditions. The location of five independent binding sites of adsorbed iodine molecules in the channels of HISL have been confirmed by single crystal diffraction,^31^ which confirms that the adsorbed iodine molecules are stabilised by a combination of strong host–guest interactions to the electron-rich pore wall and stabilised further by inter-molecular guest–guest interactions between adjacent iodine molecules with an average molecular separation of 3.7–4.1 Å. Furthermore, compared to the bare zeolite, I_2_@HISL shows eight-orders of magnitude increase in electron conductivity to 2.0 × 10^4^ S m^−1^, indicating potential in application as semiconductors. These results demonstrate that zeolites are practical candidates for I_2_ capture, and further improvements in uptake capacities will boost greatly their potential application.

### Aerogels

Aerogels are a type of mesoscale nanoporous and low-density materials comprising of assembled nano particles or polymer molecules which generate coherent pores and skeletons, which gives them their gel-like structure. Their high porosity, high surface area and excellent physical properties, such as low thermal conductivity and bulk density, promote the application of aerogels in waste removal^32^ and thermal insulation.^33^ There are a wide variety of aerogels, including oxide aerogels,^34^ chalcogenide aerogels (chalcogel),^35^ and aerogel composites.^36^

Chalcogenide aerogels have been most widely applied to the adsorption of iodine due to their high affinity to soft binding sites based upon on Pearson's hard–soft–acid–base principles. Chalcogen-based phases such as GeS_*x*_, CdSe and PbS are usually formed as aerogels using thiolysis, condensation or chemical linkage methods, and a novel aerogel denoted Cg-5C has been synthesised by mixing (CH_3_)_4_NGe_4_S_10_ and K_2_PtCl_4_ in an aqueous solution to enhance gelation.^35^ The resultant material shows a large pore volume (up to 2.3 cm^3^ g^−1^), high surface area (typically ∼1200 m^2^ g^−1^), and exhibits high iodine capacity (up to 2.39 g g^−1^). Moreover, a removal efficiency of 99% can be achieved using a flow of dry air leaving a residual iodine concentration of 4.2 ppm. Another chalcogel, denoted as ZnSnS, shows a high iodine uptake of 2.25 g g^−1^ due to its unique structural features based upon a polarisable and electron rich pore surface.^37^

Aerogel composites have also been widely investigated for iodine adsorption. Using aluminosilicate aerogels as scaffolds, Ag-based crystallites have been incorporated into the aerogel matrix *via* the wetness impregnation method.^36^ The resultant Ag-functionalised aerogel shows an iodine uptake of 0.52 g g^−1^, which is four times higher than that of the pristine aerogel. The enhancement of iodine adsorption is again due to the formation of AgI particles within the pores of the Ag-loaded material. Graphene-containing aerogels have also been successfully synthesised using hydrothermal methods.^38^ Thus, by combining a solution of graphene-oxide and aerogel a homogenous aerogel phase was formed and this shows an iodine uptake of 0.51 g g^−1^.

These studies indicate that aerogels can be utilised for iodine adsorption owing to their high uptakes. However, their amorphous structures render studies of the host–guest interactions challenging, if not impossible, which is prohibitive to the informed design of further improved materials.

### Covalent organic frameworks

Covalent-organic frameworks (COFs) are a relatively new class of porous polymers formed by the condensation of imines or boronates to form strong covalent bonds between organic building blocks. These materials show 2D or 3D open structures.^39,40^ By employing various designs and different organic linkers, a hierarchical materials system can be generated with varying structural topologies but with predictable pore size. To achieve high iodine adsorption, large pore volumes are usually beneficial due to the need for pore accessibility.

**Fig. 3:**
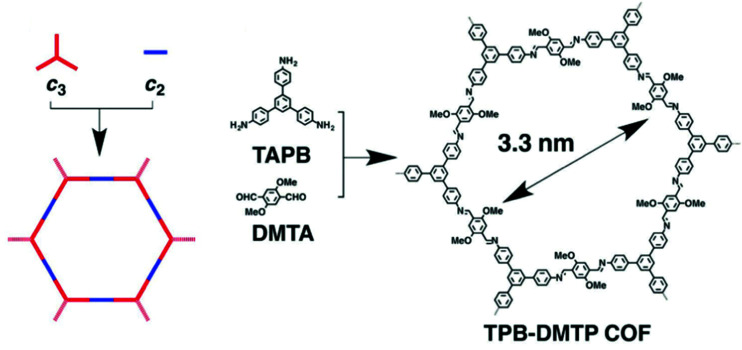
Design strategy based on the [C3 + C2] topologies for the construction of 2D hexagonal COF: TPB-DMTP. This figure has been reproduced from ref. 41 with permission from Elsevier, copyright 2018.

An imine-based COF has been synthesised by employing a [C_3_ + C_2_] topology (Fig. 3)^41^ to form hexagonal-shaped channels with a pore diameter of 3.3 nm, a surface area of 1927 m^2^ g^−1^ and pore volume of 1.28 cm^3^ g^−1^. This material shows an extremely high iodine adsorption of 6.26 g g^−1^ and shows excellent stability as no notable loss of the iodine capacity was observed after five cycles of iodine adsorption/desorption. Another imine-based COF has been designed by using a similar strategy, and the as-prepared COF also displays high and reversible iodine adsorption of 5.43 g g^−1^.^42^

A study into the effect of conjugation on the uptake of iodine in COFs has been reported as an efficient strategy to optimise these materials.^43^ The π⋯π conjugated structure in COF-LZU1 shows a higher uptake of iodine (5.3 g g^−1^) compared to the corresponding π⋯π and p⋯π conjugated structure in TpPa1 (2.45 g g^−1^) indicating that the choice of conjugated system plays a key role in iodine binding. Another recent study investigated the impact of porosity on overall iodine uptake.^44^ It was shown that a mesoporous COF, Meso-COF-3, exhibits, as expected, a higher iodine uptake than two related microporous systems, Micro-COF-1 and Micro-COF-2 (4.0 g g^−1^ compared to 2.9 g g^−1^ and 3.5 g g^−1^, respectively). However, a marked drop-off in iodine uptake was noted for extremely large pores (from 4.0 g g^−1^ to 3.3 g g^−1^) even though a higher uptake was predicted. It was rationalised that the presence of fewer adsorption interactions for iodine molecules in the highly porous COF materials was responsible for the observed reduction in iodine uptake as the porosity increases, although a degree of interpenetration or entanglement of the network might also lead to reduced uptake.

COFs tend to show higher iodine capacities compared to other interpenetrated/crosslinked networks due to the formation of wide-open channels that facilitate iodine diffusion. Moreover, variation of the organic building blocks enables the rational design for COF materials with desirable pore size, although experimental investigations on the host–guest binding mechanism in COFs remain a major challenge due to their often poor crystallinity.

### Porous organic polymers

Porous organic polymers (POPs) are constructed exclusively from organic molecules that are built up using covalent bonds. Compared to COFs, that are mainly constructed from reversible condensation reactions, the synthetic methodologies to POPs tend to be divergent and involve polycondensation,^45^ click-type reactions,^46^ trimerisations^47^ or Friedel–Crafts couplings.^48^ POP materials are generally amorphous materials that lack ordering in their structures, but they can be excellent sorbents for iodine with high uptakes.^49^ POPs can incorporate electron-rich heterocyclic N-centres, and these may greatly improve POP-iodine binding interactions.

Cyanuric chloride is a commonly used precursor and a variety of triazine-based POPs have been constructed *via* Friedel–Crafts polymerisation.^7,50,51^ A novel POP has been designed based on this strategy using cyanuric chloride as the backbone and this exhibits a high iodine capacity of 4.9 g g^−1^.^51^ Strong peaks at 170 cm^−1^ have been observed in the Raman spectrum of iodine-loaded POPs, assigned to the formation of the V-shaped pentaiodide I_5_^−^ within the pores. This suggests that charge-transfer between the guest iodine molecules and electron-rich hosts facilitates the formation of charged species, leading to the high overall iodine adsorption. Linking cyanuric chloride with triazine and triphenylamine groups affords heteroatom-rich fluorescent conjugated microporous polymer which also shows high iodine uptakes of 4.9 g g^−1^.^7^

Another strategy to construct POPs uses the Sonogashira–Hagihara cross-coupling reaction to inter-connect terminal alkyne and aryl halide groups.^52–58^ Using this methodology a series of porous aromatic framework (PAF) materials have been reported by constructing a charged tetrahedral lithium tetrakis(4-iodophenyl)borate linker with various alkyne monomers.^52^ The charged PAFs provide multiple binding sites (*e.g.*, ionic bonds and phenyl rings), which result in an iodine adsorption up to 2.76 g g^−1^. The diversity of synthetic strategies to the synthesis of POPs enables the rational design of molecular building blocks to place and control functionality within the material to maximise iodine uptake.

## Methodologies for iodine adsorption in MOFs

The adsorption of iodine into MOFs can be achieved *via* adsorption from solution^59^ or vapour diffusion,^60^ and both of these techniques are common. However, templating methods have also been used.^61^ For solution-based methods, iodine is dissolved in a nonpolar organic solvent (typically hexane or cyclohexane) and the desolvated MOFs is placed into the solution to allow the adsorption of iodine in competition with solvent molecules. In exceptional cases, a polar solvent can also be used.^62–64^ For vapour diffusion, desolvated MOFs and solid iodine are placed in a closed chamber and iodine adsorption takes place over a few hours to several days depending upon the temperature and adsorption kinetics of a given material.

**Fig. 4:**
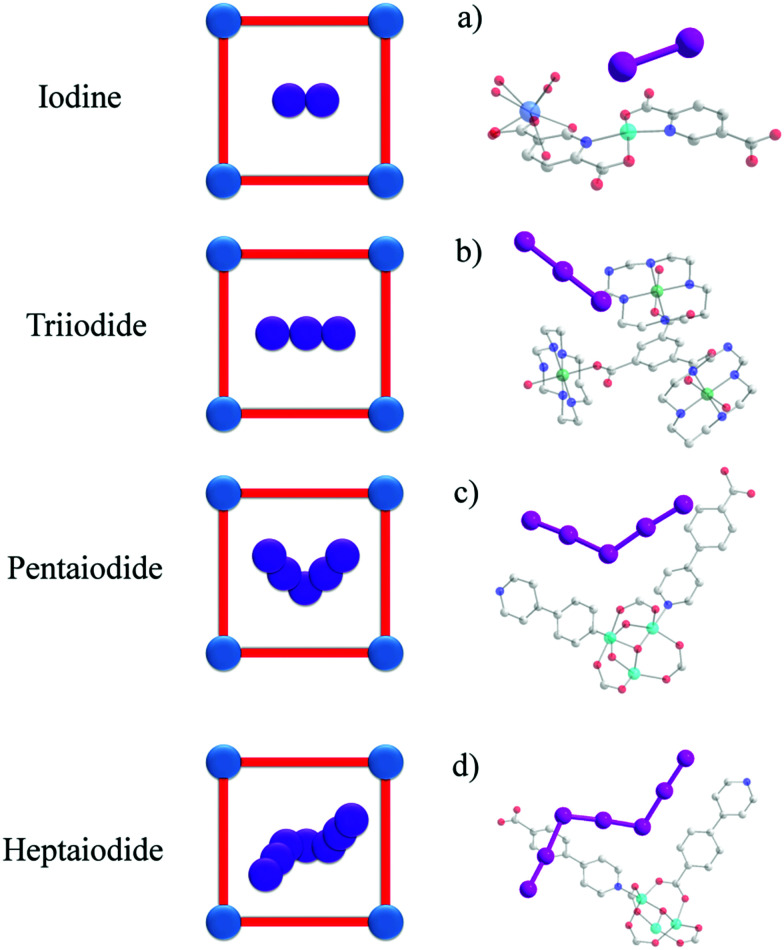
Views of the crystal structures of iodine-loaded MOFs: (a) [Sm_2_Cu_5_(OH)_2_(pydc)_6_(H_2_O)_8_]·I_8_ (H_2_pydc = 2,5-pyridinedicarboxylic acid).^67^ (b) BOF-1·(I_3_)_4_.^79^ (c and d) [Cu_6_(pybz)_8_(OH)_2_]·I_5_^−^·I_7_^−^ (pybz = 4-pyridylbenzoic acid)^85^ (C: grey, O: red, N: blue, I: pink, Ni: green, Cu: Turquoise, Gd: light blue; hydrogen atoms are omitted for clarity).

The templating procedure is less common and involves iodine being added during MOF synthesis where it acts as a structural modulator or structure-directing agent.^65^ It is a synthetic challenge to introduce iodine guests into MOFs using this technique due to the limited stability of some MOFs under these conditions, and the resultant poor crystallinity of the products makes them structurally difficult to analyse by diffraction methods. For example, an iodine-encapsulated MOF [Cu_6_(pybz)_8_(OH)_2_]·I_5_^−^·I_7_^−^ (Hpybz = 4-pyridyl benzoic acid) has been successfully synthesised using iodine as a template.^66^ The existence of both I_5_^−^ and I_7_^−^ chains within a cationic bilayer structure was confirmed by single crystal diffraction (Fig. 4). It was also noted that this structure had good stability under both acidic and alkaline solutions. Recently, a series of lanthanide-copper bimetallic MOFs, [Ln_2_Cu_5_(OH)_2_(pydc)_6_(H_2_O)_8_]·I_8_ (Ln = Sm, Eu, Gd, Tb; H_2_pydc = 2,5-pyridinedicarboxylic acid) have been synthesised (Fig. 4).^67^ In contrast to [Cu_6_(pybz)_8_(OH)_2_]·I_5_^−^·I_7_^−^, in which the polyiodides are disordered in zigzag chains, in [Sm_2_Cu_5_(OH)_2_(pydc)_6_(H_2_O)_8_]·I_8_ they form highly ordered linear chains. The complex shows good performance for photocatalytic H_2_ evolution as well as good stability under basic and alkaline solutions.

## Optimisation of MOFs for iodine adsorption

### Introduction of functional groups

**Fig. 5:**
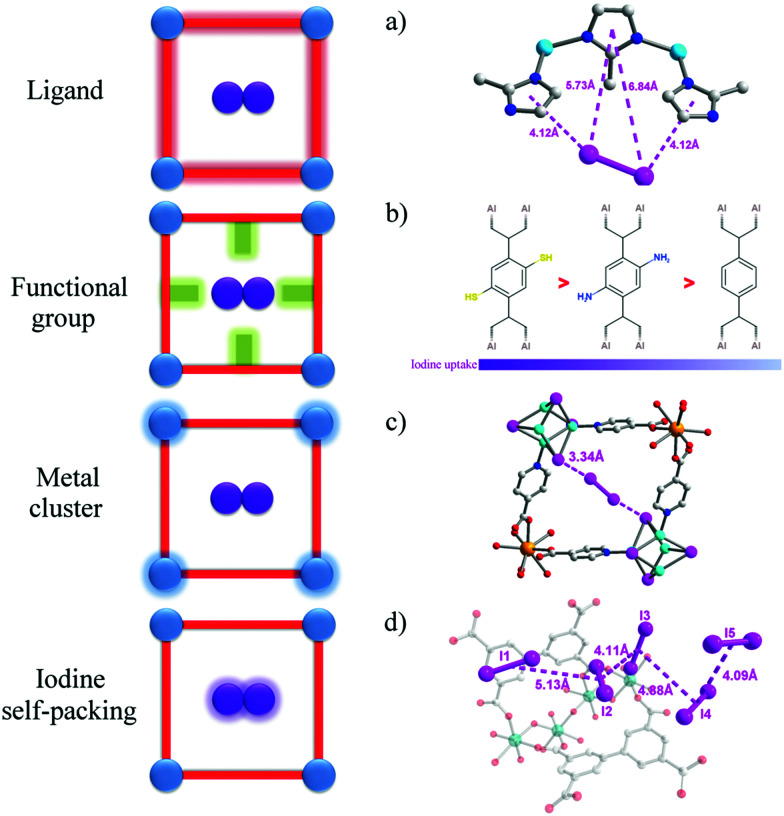
View of host–guest binding of iodine in MOFs. (a) ZIF-8·0.65I_2_^61^ (b) MIL-53-SH(Al)·0.35I_2_^69^ and MIL-53-NH_2_(Al)·0.16I_2_^68^ (c) [Tb_3_(Cu_4_I_4_)_3_(ina)_9_]_*n*_·1.5I_2_ (Hina = isonicotinic acid)^73^ (d) MFM-300(Sc)·2.62I_2_^94^ (C: grey, O: red, N: blue, I: pink, Cu: turquoise, Tb: light orange, Sc: teal, Zn: sky blue; hydrogen atoms are omitted for clarity).

The introduction of functional groups to the pores of MOFs is the most widely used strategy to increase adsorption of iodine. Molecular iodine (I_2_) is known to be an electron acceptor, so frameworks with electron-donor groups can form host–guest charge-transfer complexes, thus binding iodine molecules within the pore. Iodine sorption in derivatives of MIL-53(Al) with various functional groups [–H, –CH_3_, –NH_2_,–(OH)_2_, –COOH, –(COOH)_2_, –NO_2_, –Cl, –Br] on the benzene-1,4-dicarboxylate linker has been studied in cyclohexane solutions.^68^ These functional groups cover an extensive variety of polarities and electron-donating abilities. MIL-53(Al) adsorbs a negligible amount of iodine, while the amine-functionalised MIL-53-NH_2_(Al) shows an increase in adsorption capacity to 0.18 g g^−1^. Recently, thiol-functionalisation has been introduced to MIL-53(Al) leading to a notable improvement in iodine uptake of up to 0.33 g g^−1^ (Fig. 5).^69^ This effect is further demonstrated by two iso-reticular MOFs: {[Cd(bdc)(4-bpmh)]}_*n*_ and {[Cd(2-NH_2_bdc)(4-bpmh)]}_*n*_ (H_2_bdc = benzene dicarboxylic acid; 4-bpmh = *N*,*N*-bis-pyridine-4-ylmethylene-hydrazine).^70^ These two stable MOFs provide an excellent platform to investigate the role of amino groups on iodine adsorption, and the amino-functionalised MOF shows a two-fold increase on iodine uptake (0.38 g g^−1^) compared to the non-functionalised MOF (0.18 g g^−1^), These results confirm the role of electron-donating groups to enhance the binding and uptake of I_2_.

**Fig. 6:**
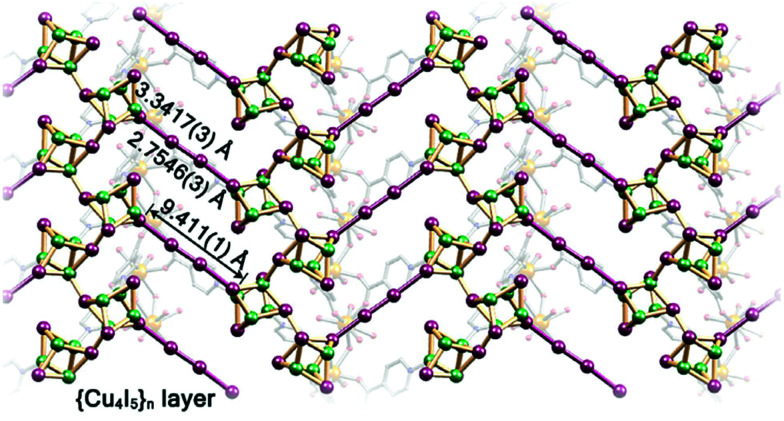
View of the linear I_4_^2−^ bridge constructed in [Tb(Cu_4_I_4_)(ina)_3_]. This figure has been reproduced from ref. 73 with permission from Elsevier, copyright 2017.

**Fig. 7:**
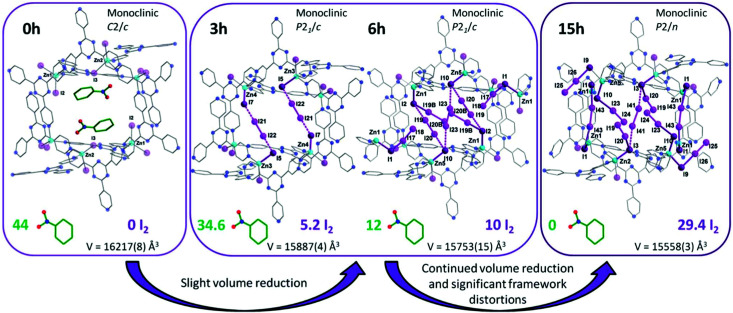
Views of X-ray crystal structure of [(ZnI_2_)_3_(TPT)_2_] (TPT = 2,4,6-tris(4-pyridyl)-1,3,5-triazine). Guest molecules of nitrobenzene are sequentially exchanged with I_2_ molecules after 3, 6 and 15 h of exposure to I_2_ vapor. This figure has been reproduced from ref. 74 with permission from The Royal Society of Chemistry, copyright 2017.

Another interesting strategy to improve iodine binding is to introduce iodide containing groups. The complex [Tb(Cu_4_I_4_)(ina)_3_(DMF)]·1.5I_2_ (Hina = isonicotinic acid) incorporates [Cu_4_I_4_] moieties and possesses channels of 9.4–9.7 Å diameter,^71^ and these are an ideal size for the assembly of I_4_^2−^ species within the structure.^71,72^ The channels thus facilitate the formation of tetraiodide anions (I_4_^2−^) *via* interactions between iodine molecules and the [Cu_4_I_4_] groups through the formation of I^−^⋯I_2_⋯I^−^ interactions with a short intermolecular distance of 3.34 Å. This result was confirmed by single crystal diffraction (Fig. 6).^73^ The interaction of iodine molecules and the framework phenyl rings has also been observed involving a I_2_⋯ring centroid interaction at of 4 Å. Owing to its high framework density, this MOF only shows a moderate iodine adsorption of 0.28 g g^−1^. Formation of a similar I_4_^2−^ assembly has been observed in activated [(ZnI_2_)_3_(TPT)_2_] (TPT = 2,4,6-tris(4-pyridyl)-1,3,5-triazine) on exposure to iodine vapour.^74^ In this material, the adsorbed iodine molecules initially form [I_4_]^2−^ moieties stabilised by interaction with accessible iodide ions from the ZnI_2_ centres within the framework. With increasing loading, [I_4_]^2−^ convert to less energetic I_3_^−^ groups that accommodate additional iodine molecules inside the pores. This system shows a high iodine capacity of 1.73 g g^−1^ (Fig. 7).

Another recent report uses acid treatment of a a Zr-based MOF, UPC-158, to increase the overall iodine uptake.^75^ The treatment involves soaking the MOF in an aqueous acidic solution (pH = 3) containing HX (X = F, Cl, Br, I) for two days. This process results in the functionalisation of the MOF with halide ions and protonated imidazolate ligands. The protonation produces different levels of fluorescence as well as changing the BET surface area and pore size, and this combination increases the iodine uptake of UPC-158 from 1.78 g g^−1^ to 2.92 g g^−1^ in the case of the HCl-treated MOF. Treatment with ethanol leads to desorption of trapped iodine molecules, but the strong interactions between iodine and the framework highlights the potential for this material to be used for long-term iodine storage.

### Shaping porosity

High surface area and porosity are key factors for the adsorption of iodine in MOFs and this typically mirrors gas adsorption in porous materials. To date, only a few MOFs with high porosity and large surface area have been reported for iodine adsorption. This is due to the poor framework stability of highly porous MOFs as the frameworks often collapse on iodine inclusion due to its caustic nature, thus preventing further investigations of cycling test.^68,75^

**Fig. 8:**
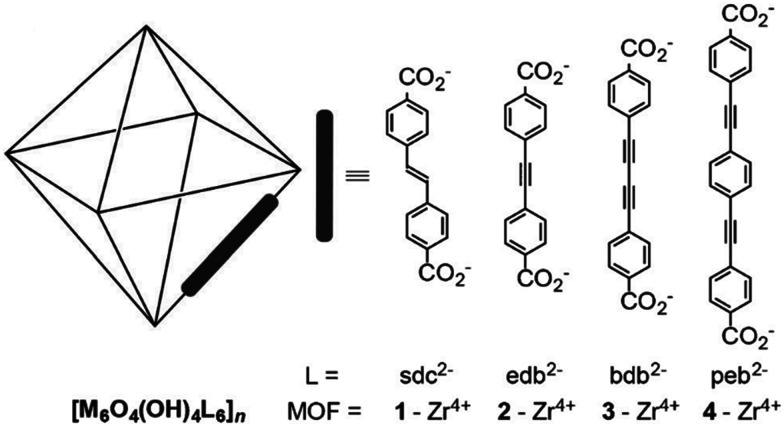
View of alkyne-functionalised ligands used in building Zr-MOFs. This figure has been reproduced from ref. 76 with permission from Elsevier, copyright 2016.

Recently, a series of iso-reticular Zr-based MOFs have been constructed using the extended form of UiO-66 where the elongated ligands contain unsaturated alkene, alkyne and units as bridges (Fig. 8).^76^ These exhibit high surface areas ranging from 2650–3850 m^2^ g^−1^, coupled with high pore volumes of 1.2–1.7 cm^3^ g^−1^. These Zr-MOFs are stable to iodine dosing with uptakes of 1.1–2.8 g g^−1^. In particular, [Zr_6_O_4_(OH)_4_(peb)_6_] [H_2_peb = 4,4′-[1,4-phenylenebis(ethyne-2,1-diyl)]dibenzoic acid] shows a pore volume of 1.16 cm^3^ g^−1^, a surface area of 2650 m^2^ g^−1^, and contains a highly elongated organic building block to give a large pore size (14.2 Å). The length of the ligand results in the formation of an interpenetrated structure that contributes to a high iodine uptake of 2.8 g g^−1^ due to the higher density of aromatic groups and metal clusters within the structure. Another benchmark MOF, HKUST-1, with a pore volume of 0.74 cm^3^ g^−1^ also shows a high iodine uptake of 1.75 g g^−1^.^77^ These values compare favourably with other benchmark solid sorbents, such as PAF-24 (2.76 g g^−1^),^52^ the conjugated microporous polymer TTPB^77^ (TTPB = triazine and triphenylamine-based fluorescent conjugated microporous polymer) (4.43 g g^−1^), and the hydrogen-bonded organic framework HcOF-1^78^ (2.9 g g^−1^).

**Fig. 9:**
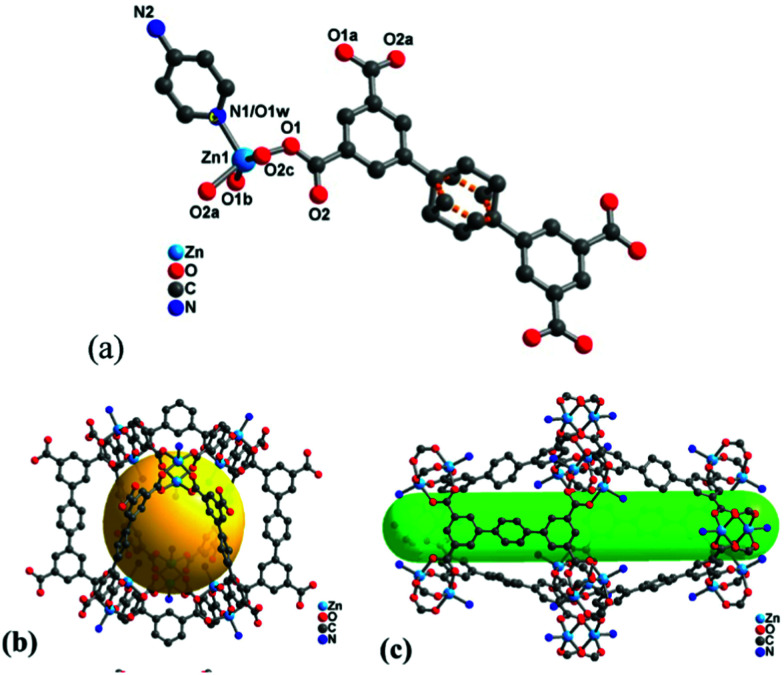
Views of the structure of [Zn_2_(tptc)(apy)] (H_4_tptc = triphenyl-3,3′′,5,5′′-tetracarboxylic acid, apy = aminopyridine); (a) the asymmetric unit, (b) small pore (9.9 Å × 17.0 Å), (c) large pore (18.8 Å × 24.7 Å). This figure has been reproduced from ref. 79 with permission from American Chemical Society, copyright 2016.

**Fig. 10:**
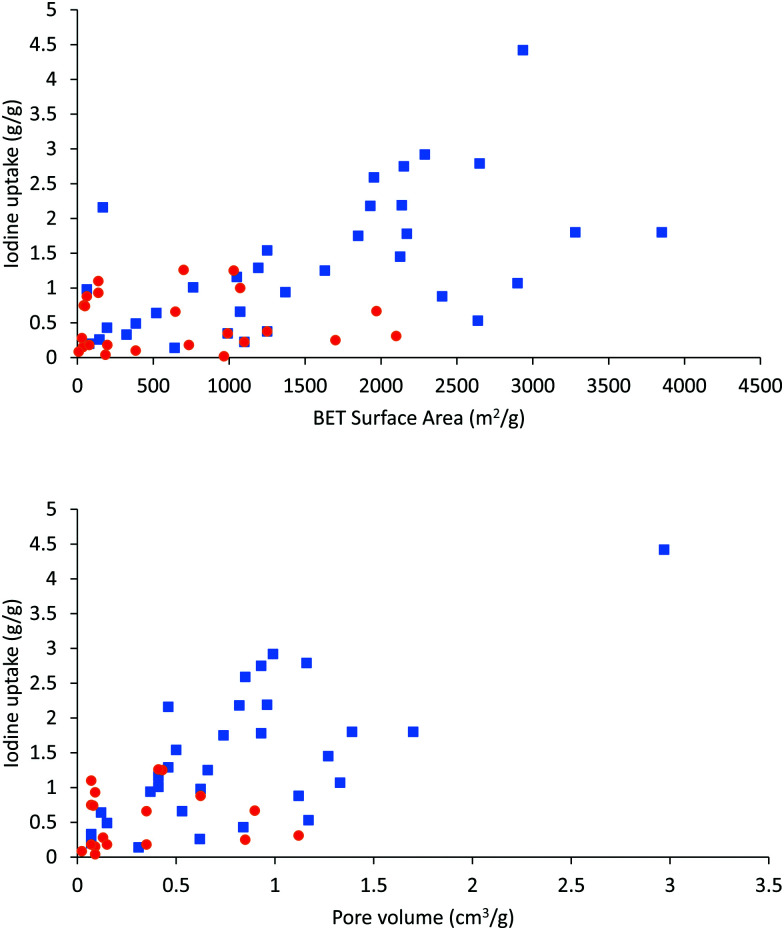
Correlations of I_2_ adsorption uptake capacity with surface area (top graph) and with pore volume (bottom graph) in host MOFs. Red and blue dots are values for adsorption *via* vapour diffusion and solution diffusion methods, respectively.

It is worth noting that high surface area and porosity are not always pre-requisites for high iodine uptake in MOFs because other factors, such as pore geometry/shape, can also affect adsorption significantly. The complex [Zn_2_(tptc)(apy)] (H_4_tptc = triphenyl-3,3′′,5,5′′-tetracarboxylic acid, apy = aminopyridine) shows a high iodine uptake (2.16 g g^−1^), albeit with a relatively low surface area (∼168 m^2^ g^−1^) and pore volume (0.46 cm^3^ g^−1^) (Fig. 9).^79^ This is due to the combined effects of the conjugated π-electron aromatic system, halogen bonding, and electron-donating amine groups. This contrasts with a thorium MOF, Th-SINAP-13, that has a significantly higher surface area of 3396.5 m^2^ g^−1^, but shows a lower iodine uptake of 0.6 g g^−1^ but with a rapid rate of adsorption rate due to its high porosity.^80^ While higher surface area and pore volume of a MOF do contribute to producing higher iodine uptakes (Fig. 10), the presence of functional groups that tailor the pore environment provide an ideal platform for iodine capture.

The overall correlation of the porosity of a MOF material with its adsorption capacity for iodine is summarised in Fig. 10, Tables 1 and 2. Vapor diffusion of iodine into MOFs with high surface areas and pore volumes generally exhibit high iodine capacities, thus affording an approximate linear relationship between the porosity and iodine capacity. There is though clearly significant scatter in these data. In particular, solution-based adsorption processes have uncertainties owing to the presence of competitive adsorption between free solvent and iodine molecules; such competitive processes require further study.

**Table 1:** Summary of iodine adsorption in MOFs *via* vapor diffusion

MOF	BET surface area (m^2^ g^−1^)	Pore volume (cm^3^ g^−1^)	Iodine uptake (g g^−1^)	Ref.
[Cd(L^1^)_2_](ClO_4_)_2_^a^	—	—	0.46	98
[Cd_3_(BTC)_2_(TIB)_2_]^a^	—	—	0.03	123
[Zn_3_(BTC)_2_(TIB)_2_]^a^	—	—	0.04	123
[Cu_2_(bitmb)_2_Cl_4_]^a^	—	—	0.31	124
{[(Me_2_NH_2_)_2_]·[Cd_3_(5tbip)_4_]·2DMF}_*n*_	—	—	1.63	125
[Ni(L^2^)_2_Cl_2_] a	—	—	0.22	106
[Ni(44pba)_2_]^a^	—	—	1.10	126
TIF-1^a^	—	—	0.54	127
[Zn(C_6_H_8_O_8_)]·2H_2_O^a^	—	—	0.16	128
[(ZnI_2_)_3_(TPT)_2_]	—	—	1.73	74
Cu-BTC	1850	0.74	1.75	77
[Cu_4_I_4_(L^3^)]	641	0.31	0.14	107
[Fe_3_(HCOO)_6_]	385	0.15	0.49	129
MIL-53-SH(Al)	324	0.07	0.33	69
Ca(sdb)	145	0.62	0.26	100
Ca(tcpb)	195	0.84	0.43	100
TMBP·CuI	520	0.12	0.64	96
ZIF-8	1630	0.66	1.25	130
[Zn_3_(dl-lac)_2_(pybz)_2_]	763	0.41	1.01	92
[Zn_2_(μ_4_-ao_2_btc)(μ-pbix)_2_]	78	0.07	0.20	131
Zn_2_(tptc)(apy)	168	0.46	2.16	79
[Zr_6_O_4_(OH)_4_ (L^4^)_6_]	2900	1.33	1.07	76
[Zr_6_O_4_(OH)_4_ (L^5^)_6_]	3280	1.39	1.80	76
[Zr_6_O_4_(OH)_4_ (L^6^)_6_]	3850	1.70	1.80	76
[Zr_6_O_4_(OH)_4_ (L^7^)_6_]	2650	1.16	2.79	76
MFM-300(Sc)	1250	0.50	1.54	94
MFM-300(In)	1050	0.41	1.16	94
MFM-300(Fe)	1192	0.46	1.29	94
MFM-300(Al)	1370	0.37	0.94	94
MFM-300(V^III^)^a^	—	—	1.42	82
MFM-300(V^IV^)^a^	—	—	1.25	82
UPC-158	2170	0.93	1.78	75
UPC-158-HF	2137	0.96	2.19	75
UPC-158-HCl	2289	0.99	2.92	75
UPC-158-HBr	2151	0.93	2.75	75
UPC-158-HCl	1954	0.85	2.59	75
SION-8^a^	—	—	0.25	132
MOF-808	1930	0.82	2.18	133
NU-1000	2126	1.27	1.45	133
MOF-867	2403	1.12	0.88	133
UiO-66	1170	0.3	1.17	134
UiO-66-FA	1705	0.73	2.25	134
UiO-67	2638	1.17	0.53	133
PCN-333(Al)	2935	2.97	4.42	86
IL@PCN-333(Al)^b^	1635	1.40	7.35	86
(ZnI_2_)_3_(tpt)_2_^a^	—	—	0.38	135
[Cd(pbica)2]·1.5DMF·2CH3OH	1073	—	0.66	136
MBM	62	0.624	0.98	137
HKUST-1@PES	1250	—	0.376	116
HKUST-1@PEI	990	—	0.348	116
HKUST-1@ PVDF	1100	—	0.225	116
Cu@MIL-101^b^	418	0.19	3.42	85
Th-SINAP-13	3396.5	—	0.60	80

a Studies were conducted on a single crystal MOF and no BET surface area or pore volume was reported.

b Study was carried out on a doped MOF.

**Table 2:** Summary of iodine adsorption in MOFs *via* solution-based processes

MOF	Solution media	BET surface area (m^2^ g^−1^)	Pore volume (cm^3^ g^−1^)	Iodine uptake (g g^−1^)	Ref.
{[WS_4_Cu_4_(4,4′-bpy)_4_][WS_4_Cu_4_I_4_(4,4′-bpy)_2_]}^b^	CCl_4_	—	—	0.20	95
[Cd_3_(BTC)_2_(TIB)_2_]_*n*_^b^	Hexane	—	—	0.16	123
[Cd(L^1^)_2_(ClO_4_)_2_]^b^	Hexane	—	—	0.19	98
TMU-16^b^	Hexane	—	—	0.22	130
TMU-16-NH_2_^b^	Hexane	—	—	1.28	130
[Zn_3_(BTC)_2_(TIB)_2_]_*n*_^b^	Hexane	—	—	0.21	123
[Zn_3_(L^2^)_2_(μ_2_-OH)_2_]_*n*_^b^	Hexane	—	—	0.28	138
[Zn_3_(L^3^)_2_(μ_2_-OH)_2_]_*n*_^b^	Hexane	—	—	0.26	138
JLU-Liu14^b^	Ethanol	—	—	0.16	139
[Cu^II^(btz)]_*n*_^b^	Cyclohexane	—	—	0.47	97
JLU-Liu32^b^	Cyclohexane	—	—	0.29	130
[Tb_3_(Cu_4_I_4_)_3_(ina)_9_]_*n*_^b^	Cyclohexane	—	—	0.28	73
TMU-15^b^	Cyclohexane	—	—	1.30	59
[Zn_7_(L^4^)_3_]_*n*_·[Zn_5_(L^4^)_3_]_*n*_^b^	Cyclohexane	—	—	0.46	140
[Cd(bdc)(4-bpmh)]_*n*_	Hexane	36	0.09	0.15	141
[Cd(2-NH_2_bdc)(4-bpmh)]_*n*_	Hexane	30	0.13	0.28	141
Cu_2_TMBD	Hexane	197	0.15	0.18	142
IFMC-10	Hexane	185	0.09	0.04	143
IFMC-15	Hexane	138	0.07	1.10	144
BOF-1	DMSO/H_2_O	138^c^	0.09	0.93	81
Cu(H_2_L^5^)	Cyclohexane	646	0.35	0.66	145
[Co(ebic)_2_]_*n*_	Cyclohexane	42^c^	0.07	0.75	146
MIL-53-NH_2_(Al)	Cyclohexane	735	0.35	0.18	68
MIL-101-NH_2_(Al)	Cyclohexane	2100	1.12	0.31	68
JLU-Liu31	Cyclohexane	1700	0.85	0.25	147
UiO-66-PYDC	Cyclohexane	1030	0.43	1.25	148
[Zn(ebic)_2_]_*n*_	Cyclohexane	50^c^	0.08	0.74	146
[Zn_2_(μ_4_-ao_2_btc)(μ-pbix)_2_]_*n*_	Cyclohexane	78	0.07	0.18	131
UiO-66	Cyclohexane	1970	0.898	0.667	148
AgNPs@UiO-66^a^	Cyclohexane	700	0.41	1.260	149
IL@PCN-333(Al)^a^	Hexane	1635	1.40	3.40	86
[DMA][In(TDC)_2_]	Cyclohexane	384.21	—	0.1	150
Th-TATAB^b^	Cyclohexane	—	—	0.075	151
{[Zn_2_(α-bptc)(H_2_O)_4_]·(pra)}_*n*_	Methanol	8.94	0.0221	0.085	152
Ag-MSHC-6^a^	H_2_O	—	—	0.077	62
[Cd(pbica)_2_]·1.5DMF·2CH_3_OH	Cyclohexane	1073	—	1.00	136
MIL-125-NH_2_@chitosan	H_2_O	965.8	—	0.019	63
MBM	H_2_O	62	0.624	0.88	137
HKUST-1@PES	Cyclohexane	1250	—	0.376	116
HKUST-1@PEI	Cyclohexane	990	—	0.348	116
HKUST-1@ PVDF	Cyclohexane	1100	—	0.225	116
Ag_2_O-Ag_2_O_3_@ZIF-8^a^	H_2_O	369.9	0.14	0.23	153
Ag@MIL-101^a^	H_2_O	1045	1.54	2.14	84

a Study was carried out on a doped MOF.

b Studies were conducted on a single crystal MOF and no BET surface area or pore volume was reported.

c Langmuir surface area since BET surface areas of these MOFs were not reported.

### Redox metal centres

Studies on MOFs with redox-active metal centres for iodine adsorption have been rarely reported due to the very limited number of redox-active MOFs and the limited framework stability upon iodine inclusion. A representative example is BOF-1, constructed from benzene-1,3,5-tricarboxylate and Ni(ii) centres. This material reacts with iodine to produce a positively-charged framework containing Ni(ii/iii) ions with I_3_^−^ and I_2_ species in the channels.^81^ In this system, two thirds of the Ni(ii) ions incorporated in the framework are oxidised to low spin Ni(iii) centres upon exposure to iodine, as confirmed by magnetic susceptibility measurements. BOF-1 shows an iodine uptake of 1.03 g g^−1^ in the form of I_3_^−^ and I_2_. Four independent I_3_^−^ positions are found to act as counter anions within the pores of the positively-charged framework, and the intermolecular distances between the triiodides are *ca.* 4.6 Å. This unprecedented redox reaction between iodine and the host framework was attributed to the ability of the framework to stabilise the Ni(iii) oxidation state.

Another example uses redox-active vanadium centres in MFM-300(V^III^) for iodine adsorption.^82^ MFM-300(V^III^) shows a reversible uptake of iodine of 1.42 g g^−1^, with the adsorbed iodine molecules binding in two domains to form helical chains within the MOF. Interestingly, the adsorption of iodine results in an increase in conductivity by a magnitude of 10^6^, and this makes MFM-300(V^III^) an excellent candidate for detecting iodine. The increase in conductivity is caused by the host–guest charge transfer interactions as a result of the partial oxidation of V^III^ to V^IV^ and the formation of I_3_^−^ within the pore. The presence of I_3_^−^ was confirmed using a combination of X-ray photoelectron spectroscopy and Raman spectroscopy.

### Doping

Doping of MOFs with metals and active molecules is frequently used in catalysis.^83^ Doping of MOFs with metal centres has been shown to increase the iodine uptake *via* the formation of metal iodides, as observed for aerogels. An example of this uses MIL-101 doped with Ag(ii) ions.^84^ Varying levels of Ag ions can be doped into MIL-101 *via* soaking in solution, and the subsequent uptake of iodide was reported to be 0.24 g g^−1^ when loaded with 25% Ag(ii), 12 times higher than that of bare MIL-101. This methodology was confirmed to work also in aqueous solutions, demonstrating its potential for removal of iodine from water. A similar process using copper nanoparticles deposited inside MIL-101 can successfully trap iodine to an uptake of 3.42 g g^−1^.^85^

PCN-333(Al) can be doped with an ionic liquid to give an iodine uptake of 7.35 g g^−1^ from vapour and 3.4 g g^−1^ from hexane solutions.^86^ These results are some of the highest recorded uptakes in vapour or solution, and originate from interactions between iodine and the halide, in this case bromide present in the ionic liquid located inside the pores of the MOF. This technique was replicated with MIL-101(Cr) to produce an increase in iodine uptake from 0.39 g g^−1^ to 0.96 g g^−1^ on doping with an ionic liquid.

### Physical retention of iodine

#### Pressure-induced amorphization

**Fig. 11:**
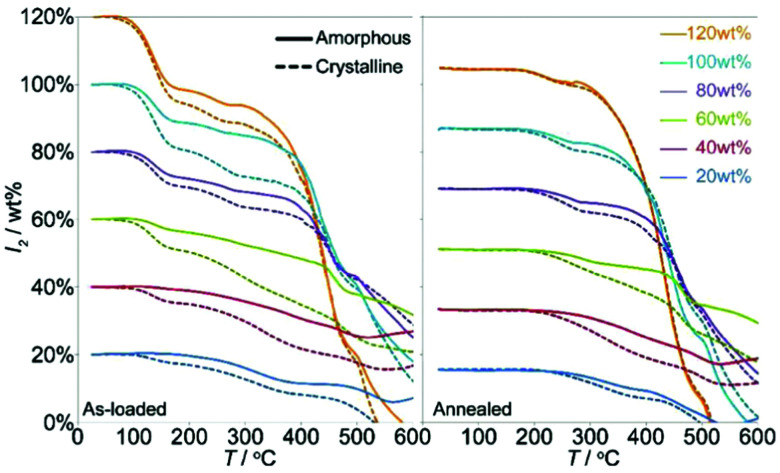
The mass loss associated with I_2_ release from the crystalline and amorphized ZIF-8 based by TGA of the as-loaded (left) and annealed (right) samples. This figure has been reproduced from ref. 87 with permission from the American Chemical Society, copyright 2011.

For solid-state adsorption of radioactive iodine, it is important to trap the iodine within the pores of the framework and eliminate the potential for surface-adsorbed iodine to be released post-sorption to the atmosphere, for example, during transport. A method denoted as “pressure-induced squeezing” has been developed to increase the retention of iodine by distorting the morphology of the MOF host material.^87^ ZIF-8, which shows an iodine adsorption capacity of 1.25 g g^−1^, was selected for a proof-of-concept test. The as-prepared iodine-loaded ZIF-8 powder was compressed into extruded pellets under a pressure of 1.2 GPa. During this process, the host–guest system undergoes amorphisation as measured by pair density functional (PDF) analysis, and this confirmed that the short-range order of the host–guest system was retained. Thermal gravimetric analysis (TGA) confirmed that the compressed pellets are sufficiently deformed to kinetically trap iodine within the framework pores, thus eliminating the release of surface-adsorbed iodine (Fig. 11).

A systematic study was conducted to further investigate the pressure-induced process using a series of ZIFs (ZIF-4, ZIF-69, ZIF-mnIm) as hosts.^88^ By ball-milling, all of these sorbents experience a similar amorphisation process as the ZIF-8 material on iodine adsorption, but they maintain the short range order of their structures. ZIF-mnIm exhibits the highest level of retention of iodine (up to 0.25 g g^−1^) of the three tested samples, and this stems from the methyl-functionalisation controlling and hindering loss of adsorbed iodine species. This simple mechanical modification provides new insights for control of nanoscale sorption, opening up possibilities for future applications in interim storage and controlled release of radioactive iodine and other substrates.

#### Glass sintering

Iodine retention using storage on a geological timeframe may be the best current option for the disposal of such radio-active nuclides. In this context the adsorption of iodine in MOFs can also be achieved *via* glass sintering.^12^ A combination of ZIF-8 and HKUST-1 with sintered glass and metallic silver flakes has been converted to a glass-composite material (GCM) for iodine capture.^89^ A uniform monolith was formed upon sintering with no loss of iodine uptake, as confirmed by TGA. The formation of AgI was observed during the heating process. The sintered material shows excellent thermal and chemical stability and, most importantly, passes the product consistency test,^90^ a standard test for radioactive waste forms. These properties demonstrate the potential of immobilising radioactive iodine in MOF materials for safe transport and storage.

## Characterisation of iodine-loaded MOFs

### UV/Visible spectroscopy of MOFs upon iodine adsorption

UV/Vis spectroscopy is often used to measure the removal of iodine from solution by monitoring the characteristic absorption peak at *λ* ≈ 220 nm. This, for example, has been used in a comprehensive investigation into the effect of different functional groups on the iodine uptake with Th-UiO-66.^91^ It was confirmed that electron donating groups such as NH_2_, –Cl, –OH and –Br improved both the rate and quantity of iodine capture from a cyclohexane solution. It was also observed that the disubstituted Th-UiO-66 MOFs had lower removal rates for iodine compared with their monosubstituted counterparts. This was attributed to changes in the conjugated π-electron density and the shape of the pore. These results correlate well with data for functionalised MIL-53(Al) as both achieve maximum removal efficiency when amine groups are incorporated.^69^ Differences are observed though on incorporation a methyl group into these MOFs with a decrease for Th-UiO-66-CH_3_ but an increase observed for MIL-53(Al)-CH_3_ in the rate of removal of iodine from solution. This difference in behaviour was attributed to differences in pore volumes and aperture sizes, highlighting the importance of shape as well as functionality in the capture of iodine.

**Fig. 12:**
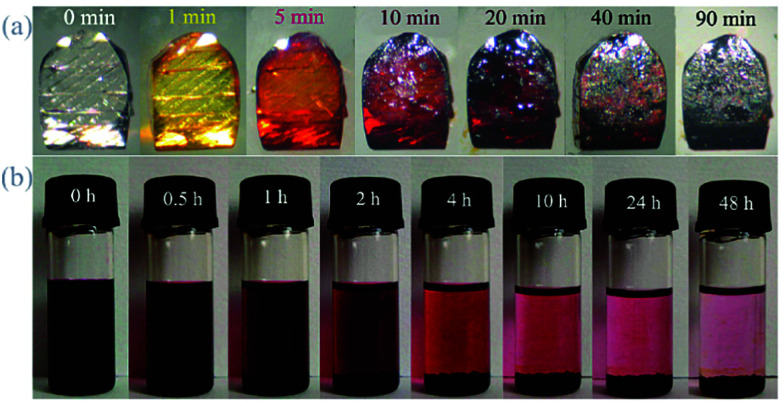
(a) Visual color change of single crystals of [Zn_3_(dl-lac)_2_(pybz)_2_] when immersed in iodine containing cyclohexane solution (0.1 M L^−1^). (b) Progress of I_2_ enrichment of crystals of I_2_@[Zn_3_(dl-lac)_2_(pybz)_2_] in cyclohexane solution. This figure has been reproduced from ref. 92 with permission from the American Chemical Society, copyright 2010.

The colour of MOFs typically darkens on uptake of iodine molecules. The gradual colour change of [Zn_3_(DLlac)_2_(pybz)_2_]_*n*_ (H_2_dl-lac = lactic acid, Hpybz = 4-pyridylbenzoic acid) on immersion into an iodine–cyclohexane solution has been investigated in detail (Fig. 12).^92^ The adsorbent changed from colourless to brown with concomitant change of the dark brown solution to pale red, consistent with iodine is being trapped by the MOF host. UV/Vis spectroscopy was used to monitor the concentration of iodine in solution to calculate the concentration of iodine adsorbed by the MOF together with the kinetics of iodine uptake and release. The release of iodine from I_2_@[Zn_3_(DLlac)_2_(pybz)_2_]_*n*_ in ETOH was then followed spectroscopically and confirmed that the release takes place linearly over time. The release of iodine is governed by the homogenous host–guest interaction; however, I_2_@[Zn_3_(DLlac)_2_(pybz)_2_]_*n*_ requires more than 11 days to reach equilibrium. This is significantly longer than zeolite 13× and commercial activated carbons, which typically take only a few hours to reach equilibrium.

### Quantification of iodine adsorption in MOFs

Thermal gravimetric analysis (TGA) is commonly employed to quantify the amount of iodine adsorbed in MOFs. Coupling this with mass spectrometry (TGA-MS) can uncover useful information by providing the precise mass of the evolved components as a function of temperature. Mass spectrometry uses multiple ion detection to detect selected masses produced during the experiment, for example confirming the percentage weight loss observed on release of ^127^I and ^254^(I_2_).^93^

A gravimetric method^76^ can also be used to measure iodine uptakes in materials that chemisorb iodine or have low thermal stability. This method has the advantage of being non-destructive and can be used to investigate kinetics of adsorption. A similar method to adsorption of iodine from vapour is used, and the MOF sample is removed for weighing at set times. The change in mass upon iodine adsorption is then compared to the mass of the original sample to calculate the total iodine uptake. The results plotted over time clearly present the rate at which iodine is adsorbed and the point of saturation. The level of iodine uptake can also be confirmed using elemental analysis.

**Fig. 13:**
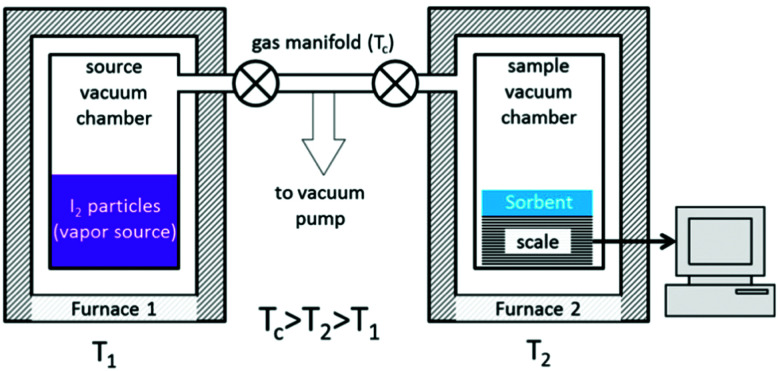
Schematic view of an iodine adsorption unit. This figure has been reproduced from ref. 93 with permission from the American Chemical Society, copyright 2017.

Another method to quantify iodine adsorption is to record the sorption isotherm of iodine from the vapour phase. Whilst this is a conventional technique for volatile gases (*e.g.*, CO_2_, CH_4_, N_2_), it is highly challenging to measure isotherms for iodine uptake due to the need to control the pressure of iodine vapour at a given temperature. A purpose-built rig has been developed to enable *in situ* measurements of iodine uptake (Fig. 13).^94^ This apparatus is built from standard stainless steel and nickel sealing gaskets as protection from the highly reactive iodine. The whole system can be retained at 120 °C to avoid condensation of iodine, while the target pressure of vapour can be controlled accurately by heating the iodine reservoir at various temperatures to dial-up the appropriate vapour pressure. A study of a series of nanoporous materials, including activated charcoals, zeolites and MOFs, has been reported using this system and good agreement with previously reported results was obtained.^94^ For example, an iodine uptake of 0.16 g g^−1^ was observed for the silver-containing zeolite mordenite [Ag(i)-MOR] at low pressure (*P*/*P*_0_ = 0.1). This was attributed to the strong interaction between iodine and Ag(i) ions through a chemisorption process to form AgI clusters within the pores. Very low additional iodine uptake was observed beyond *P*/*P*_0_ = 0.4 owing to the relatsively small pore size and surface area of this zeolite. In contrast, negligible amounts of iodine vapour were adsorbed below a relative pressure of *P*/*P*_0_ = 0.3 in two benchmark MOFs, ZIF-8 and HKUST-1, indicating the absence of any chemisorption process with either material. Beyond this pressure, a gradual increase in adsorption of iodine was observed as a function of pressure, and the final adsorption equilibrium was achieved at 1 bar reflecting a physisorption process. This contrasts with the chemisorption process observed for Ag(i)-doped zeolites; MOF systems rely typically upon weak and long-range iodine-framework supramolecular interactions, while doped Ag(i)-MOR samples exhibit strong interactions *via* chemisorption.

### Direct visualisation of binding domains for adsorbed iodine molecules

The direct visualisation of the preferred binding domains for adsorbed iodine in host materials gives important understanding of the host–guest interactions that drive adsorption processes. The crystalline nature of MOFs allows advanced diffraction studies to be undertaken; such studies can be more problematic for non-crystalline adsorbents such as chalcogels^6^ and organic polymers.^7^ However, determination of the location of adsorbed iodine molecules in a crystalline matrix can be complicated by the potentially high activation barrier for diffusion of iodine into and within the host structure. This can result in significant disorder of the structure leading to diffuse X-ray scattering rather than sharp Bragg peaks.^75,85^ A few strategies including both single crystal diffraction^73,74,81,95–100^ and high resolution powder X-ray diffrection (PXRD)^60,77,93^ have been adopted involving careful control of iodine loading and use of a synchrotron source. In some cases, the location of adsorbed iodine molecules has been determined successfully determined (Fig. 5).

Single crystal diffraction is the most straightforward method to monitor phase changes of host materials on iodine adsorption. The introduction of functional groups into porous MOFs is an important approach to tune the iodine capacity by providing additional binding sites. To date, only a few crystal structures have been reported to confirm the formation of X–I⋯I–I⋯I–X complexes (X = Zn, Cu), as discussed above.^73,74,94^ An unusual system built around W and Cu clusters and 4,4′-bpy ligands within a diamond-type network^89^ shows trapped iodine molecules associated with the coordinated iodide group on the MOF host. The adsorbed iodine molecules are accommodated between the iodide ions of adjacent bridging clusters to form the polyiodide I_4_^2^^−^ anions which lie parallel to the 4,4′-bpy bridges. Many reported crystal structures of iodine-loaded MOFs exhibit host–guest interactions between iodine molecules and the aromatic rings in the organic bridging ligands. For example, [Co_1.5_(bdc)_1.5_(H_2_bpz)] (H_2_bdc = 1,4-benzenedicarboxylic acid, H_2_bpz = 3,3′,5,5′-tetramethyl-4,4′-bipyrazole) shows two types of rectangular 5.7 × 3.2 Å and 5.7 × 4.5 Å channels.^99^ It was confirmed that adsorbed iodine molecules reside linearly within these channels with I⋯H–C interactions to the phenyl –CH groups on the channel walls observed. A I⋯H contact of 3.14 Å suggests a significant host–guest interaction in this system. The calcium-based MOF, SBMOF-2, comprises of isolated CaO_6_ octahedra bridged by 1,2,4,5-tetrakis(4-carboxyphenyl)benzene linkers.^100^ The resultant channels are decorated with phenyl rings which act as sorption sites for iodine molecules. Indeed, the trapped iodine molecules are highly ordered and point to the centre of the phenyl ring with a short I⋯phenyl ring distance of 3.47 Å. This is supplemented by additional hydrogen bonding (I⋯H = 3.35 Å), thus providing key crystallographic evidence for the mechanism of host–guest binding in this system.

Recently, a family of highly rigid and iso-structural MOFs, MFM-300(M) (M = Al, Sc, Fe, In) has been reported to show high iodine uptakes (0.94–1.54 g g^−1^), with MFM-300(Sc) showing the highest uptake (1.54 g g^−1^).^93^ Advanced structural analysis using synchrotron radiation confirms the presence of intermolecular interactions between iodine molecules (I_2_⋯I_2_ distances of ∼3–4 Å), resulting in the formation of aggregated iodine chains within the pores of the MOF. The disordered iodine chains in the MFM-300 materials afford a high iodine packing density of 3.08 g cm^−3^, ∼63% of that of solid iodine (4.93 g cm^−3^ at 298 K). The combination of suitable pore size (6–8 Å), shape/geometry of channels and functional groups (*i.e.*, pendant hydroxyl bridges) provide a unique platform to induce and stabilise the formation of a complex assembly of molecular iodine, resulting in highly efficient packing and exceptional storage density.

Another important technique to investigate iodine-loaded materials is synchrotron X-ray pair distribution function (PDF) analysis.^101,102^ This collects total scattering data to give structural information within the local region of the host–guest system. Differential (d-) PDF enables the study of species inside a nanoporous framework (*e.g.*, I_2_ in ZIF-8) by subtraction of data for the framework from data for the substrate-loaded framework.^30,60,77,87,93^ For example, d-PDF analysis [augmented by density functional theory (DFT), Grand Canonical Monte Carlo (GCMC) analysis] has been used to understand the occupancy of iodine sites within the ZIF-8 framework;^60^ this has enabled the Rietveld refinement of the corresponding X-ray diffraction data.^60^ ZIF-8 possesses two type of cages: the small cage constructed from four-membered rings are too constrained to allow diffusion of guest iodine molecules, while the larger cage of 11.6 Å in diameter is connected by six-membered rings and accommodates adsorbed iodine molecules. Two independent binding sites were located, both of which are in the middle of the pore to form a specific host–guest interaction between iodine and the HmIm (HmIm = 2-methylimidazole) linkers of ZIF-8, with a I_2_⋯imidazole distance of 4.12 Å (Fig. 5). By subtracting the reference PDF data measured for pristine ZIF-8, a differential analysis of I⋯I and I⋯framework interactions were obtained. With changes of peak intensity and positions, the incremental d-PDFs provide detailed insights into the process of adsorption and binding of iodine within the pore.

PDF analysis has also been used to confirm that the local order of HKUST-1 was preserved on adsorption of iodine, although loss of Bragg peaks was clearly observed.^77^ From the analysis, the characteristic peaks in the PDF data correlated with framework Cu–O (∼2 Å) bonds and guest I_2_⋯I_2_ (∼2.7 Å) interactions. Moreover, it enabled the quantification of the ratio of Cu/I in the iodine-loaded system, which was found to be in good agreement with the results obtained from TGA analysis.

### Spectroscopic analysis of iodine-loaded MOFs

Raman, UV/Vis and X-ray photoelectron (XPS) spectroscopy can be used to detect iodine species. Notably, XPS enables not only the determination of the elemental composition, but also the electronic state of elements within the material. Theoretically, the binding energy for the 3d_5/2_ orbital of iodine is 620.1 eV and is accompanied by another characteristic peak for the 3d_3/2_ orbital at 630.6 eV. The interaction between iodine molecules and the framework can promote shifts of the binding energies for 3d_5/2_ and 3d_3/2_ orbitals on adsorption. For example, the presence of iodine–iodide interactions within [Cu_6_(AcNTB)_6_(ClO_4_)_3_(H_2_O)_*n*_]·3I_3_ {HAcNTB = *N*-[*N*′-(carboxymethyl)benzimidazole-2-ylmethyl]-*N*,*N*-bis(benzimidazole-2-ylmethyl)amine} was confirmed using XPS.^103^ It was observed that the characteristic peak for iodine was not of a symmetric Gaussian form, but comprised of two peaks at binding energies (BE) of 619.1 and 620.8 eV assigned to I^−^ and I_2_, respectively. This confirmed dissociation of adsorbed iodine in this system.

Vibrational spectroscopy has been used widely in the analysis of iodine-adsorbed MOFs. The I–I vibration is Raman active with a distinct band at 180 cm^−1^ for solid iodine. The interaction of adsorbed iodine and the framework polarises the I–I bond and leads to blue shifts of the Raman band of typically 5–15 cm^−1^.^68,93^

## Computational investigations

**Fig. 14:**
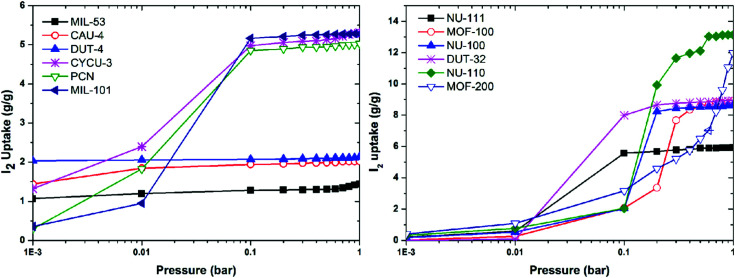
Simulated iodine adsorption isotherms at 298 K for selected MOFs. This figure has been reproduced from ref. 104 with permission from Elsevier, copyright 2014.

Computational studies, including density functional theory (DFT), Grand Canonical Monte Carlo (GCMC) and molecular dynamics (MD) calculations and simulations can be used to analyse iodine-adsorbed MOF systems. In particular, these methods enable the prediction of both the quantity of iodine that can be adsorbed and their general location *via* location of electron density of molecules within the pore. This review primarily focuses on the discussion of experimental findings on iodine adsorption in MOFs and thus computational studies are only introduced briefly here.^60,93,104,105^ Recently, a group of twelve MOFs with diverse surface areas and pore volumes have been screened by GCMC modelling using standard universal force fields.^104^ It was confirmed that MOFs of high pore volume and surface area are favoured for iodine storage under ambient conditions. Of these NU-110 (surface area of 7140 m^2^ g^−1^, pore volume of 4.4 cm^3^ g^−1^) shows the highest calculated iodine capacity of up to 11 g g^−1^ (Fig. 14). A systematic GCMC/DFT study on the ZIF series materials for iodine adsorption has also confirmed that high surface areas and large metal–ligand cages can effectively increase the adsorption capacity of the material for iodine.^105^ It was confirmed that polar functional groups lead to enhanced iodine adsorption, while the presence of water can have a reverse effect and hinders iodine uptake.

## Iodine-loaded MOFs as composite materials

### Heterogeneous catalysis

Recently, iodine-loaded [Ni(L)_2_Cl_2_] [L = 1,1′-(9,9-dimethyl-9*H*-fluorene-2,7-diyl)bis(1*H*-1,2,4-triazole)] has been reported as a heterogeneous catalyst for the silylcyanation of aromatic aldehydes under mild conditions.^106^ High catalytic activity with up to 76% conversion and reusability after 5 cycles were reported. Similarly, iodine-loaded Zr(DMBD) (H_2_DMBD = 2,5-dimercapto-1,4-benzenedicarboxylic acid) acts as an efficient iodination agent for 2-ethynylbenzyl alcohol with a 93% conversion without any by-product detected.^96^ Iodine-loaded Cu_4_I_4_L [L = 5,5′,5′′-(2,4,6-triethylbenzene-1,3,5-triyl)tris(2-(pyridin-3-yl)-1,3,4-oxadiazole)] has been tested as a heterogeneous catalyst for Friedel–Crafts alkylation. Scanning electron microscopy (SEM) was used to establish that the morphology of the MOF was well-preserved after five catalytic cycles,^107^ confirming the potential recyclability of such composite materials in catalytic reactions. These studies confirm the potential for the development of iodine-loaded MOFs as heterogeneous catalysts.

### Sensing of iodine using MOFs

#### Resistance sensing

**Fig. 15:**
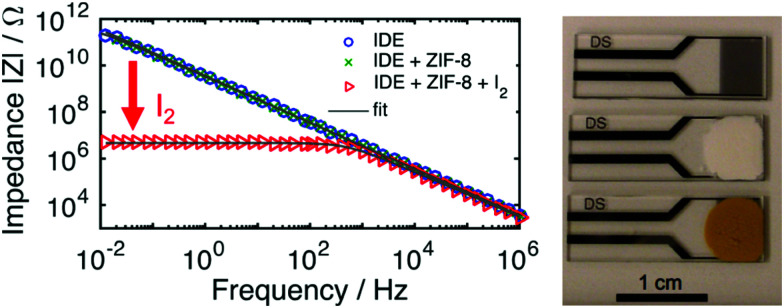
Impedance response for sensing applications. (left) Non-MOF coated IDE with high impedance (|*Z*| > 10^11^ Ω at 10 mHz) and highly capacitive character (*θ* ≈ −90°). In a thin film of ZIF-8, the low frequency impedance phase angle barely changes. Upon exposure to I_2_ gas in air, a large change is produced for both the impedance and phase angle at low frequencies. (right, top to bottom) IDE, MOF film on IDE, I_2_@MOF film on IDE. This figure has been reproduced from ref. 108 with permission from the American Chemical Society, copyright 2017.

The enhanced conductivity, and thus the reduced resistance, observed on iodine adsorption in porous MOFs has enabled development of direct electrical read-out sensors based upon the high selectivity for binding of iodine gas.^108^ ZIF-8 has a high iodine uptake under ambient conditions, and a thin film of the MOF has been deposited onto silica mounted on a Pt-based interdigitated electrode (IDE) (Fig. 15). Impedance spectroscopy was then used to directly detect the adsorption of iodine in real-time. Iodine was readily detected at 25 °C in air within 720 s of exposure. When the sensor was run at 70 °C in air, a >10^5^ times decrease in the resistance of the thin film was observed on adsorption of 116 wt% iodine. No observable interference was produced by competing gaseous molecules present in the air, such as H_2_O, O_2_, Ar, CO_2_, and methanol. A resistance-based sensor has also been developed using the MFM-300 materials.^109^ In this study, the choice of metal ion that constitute the MOF was shown to effect the response and reversibility of the sensor. MFM-300(Al) and MFM-300(In) produced larger changes in resistance after each cycle compared with MFM-300(Fe).

#### Conductivity sensing

The electrical conductivity of porous materials can be enhanced by loading with iodine molecules, suggesting that they could have potential for use as electrical sensors (Table 3). Different mechanisms have been reported for the enhancement of conductivity,^110^ including the interaction between iodine and the ligand, metal centre or metal cluster.^71,92,111^ With a focus on environmental monitoring, the development of portable sensors enables the direct electrical detection of gaseous iodine under ambient conditions using facile fabrication techniques and commercially available materials.

**Table 3:** Summary of electrical conductivities for iodine-loaded MOFs

MOF	Conductivity of bare MOF (S cm^−1^)	Conductivity of iodine-loaded MOF (S cm^−1^)	Conductivity enhancement (*x*)	Ref.
Cu[Ni(pdt)_2_]	1 × 10^−8^	1 × 10^−4^	∼10^4^	111
[Cu_6_(pybz)_8_(OH)_2_](I^−^)_2_	8.04 × 10^−9^	8.11 × 10^−7^	∼100	66
[Co_1.5_(bdc)_1.5_(H_2_bpz)]	2.59 × 10^−9^	1.56 × 10^−6^	∼1000	99
[Co(ebic)_2_]_*n*_	2.46 × 10^−9^	2.21 × 10^−7^	∼90	146
[Eu(L^1^)]	8.27 × 10^−7^	2.71 × 10^−5^	∼33	154
IFMC-15	2.59 × 10^−9^	2.07 × 10^−7^	∼80	144
{[(Me_2_NH_2_)_2_]·[Cd_3_(5-tbip)_4_]}_*n*_	1.71 × 10^−8^	1.30 × 10^−6^	∼76	125
MET-3	0.77 × 10^−4^	1 × 10^−3^	∼13	155
[Tb_3_(Cu_4_I_4_)_3_(ina)_9_]_*n*_	5.72 × 10^−11^	2.16 × 10^−4^	∼10^8^	73
[Zn_3_(DL-lac)_2_(pybz)_2_]_*n*_	—	*σ* ^‖^ = 3.4 × 10^−3^	—	92
		*σ*_⊥_ = 1.7 × 10^−4^		
[Zn(ebic)_2_]_*n*_	4.33 × 10^−9^	3.47 × 10^−7^	∼80	146
Mn(F_4_TCNQ)(py)_2_	5 × 10^−10^	1.4 × 10^−4^	∼10^5^	156
MFM-300(V)	1.7 × 10^−10^	1.16 × 10^−4^	∼10^6^	121

A two orders of magnitude increase in electrical conductivity was observed on loading [Zn_3_(dl-lac)_2_(pybz)_2_] (H_2_dl-lac = lactic acid, Hpybz = 4-pyridylbenzoic acid) with iodine, and this has been attributed to the interaction between the iodine and the aromatic rings of the ligands.^92^ More interestingly, introduction of iodine molecules into the redox-active Cu[Ni(pdt)_2_] (H_2_pdt = pyrazine-2,3-dithiolate) results in an ∼10 000 fold increase in electrical conductivity.^111^ Very recently, [Tb(Cu_4_I_4_)(ina)_3_(DMF)] (Hina = isonicotinic acid) has been reported to show seven orders of magnitude enhancement in conductivity ongoing from the pristine (5.72 × 10^−11^ S cm^−1^) to the iodine-loaded material (2.16 × 10^−4^ S cm^−1^).^73^ This dramatic increase was assigned to the presence of I^−^⋯I_2_⋯I^−^ interactions inside the pore, as confirmed by single crystal diffraction studies (Fig. 6).

A single crystal iodine sensor based upon HKUST-1 has also been developed.^112^ Though there is an initial report of MOF crystallized films in direct electrical readout sensors,^167^ most require the prepartion of a powder sample for use in the sensor. In this case the powder must be compressed, which can cause structural collapse or framework amorphisation that can affect the reproducibility of results between batches of samples. The single crystalline sample produced consistent and reproducible results; however, it can still be challenging to produce single crystals of MOFs that are stable to iodine.

## Summary and outlook

The complexity and variability of framework structures and their compositions play a dominating role in their ability to adsorb specific gases and substrates. This is also the case for iodine adsorption as a wide range of iodine uptakes is observed for both vapour-based (Table 1) and solution-based studies (Table 2). The practicality of using MOFs as radioactive waste traps has also been investigated by monitoring the stability of the MOF with respect to iodine adsorption and desorption (Table 4), as well as investigating methods to produce a stable waste forms that do not leach iodine into water.^12^ Materials that trap iodine irreversibly within a MOF structure make them ideal candidates for long-term iodine storage.^76^

**Table 4:** Stability of reversibility results of selected MOFs with high iodine uptake (>1.0 g g^−1^)

MOF^a^	Iodine uptake (g g^−1^)	Stability	Reversibility	Ref.
[Zn_3_(dl-lac)_2_(pybz)_2_]	1.01	Stable	Fully reversible	92
[Ni(4,4′-pba)_2_]	1.10	Stable	Fully reversible	126
IFMC-15	1.10	Stable	Fully reversible	144
ZIF-8	1.25	Stable up to 0.7 g g^−1^	No	60
UiO-66-PYDC	1.25	Stable	Fully reversible	148
TMU-16-NH_2_	1.28	Stable	Fully reversible	130
TMU-15	1.30	Stable	Fully reversible	59
MFM-300(Sc)	1.54	Stable	Fully reversible	94
[(ZnI_2_)_3_(TPT)_2_]	1.73	Stable	N/A	74
CuBTC	1.75	Stable	Fully reversible	77
Zn_2_(tptc)(apy)	2.16	Stable	Fully reversible	79
[Zr_6_O_4_(OH)_4_(peb)_6_]	2.79	Stable	No	76

a H_2_dl-lac = lactic acid; Hpybz = 4-pyridylbenzoic acid; H44pba = 4-(4-pyridyl)benzoic acid; H_2_pydc = 2,5-pyridinedicarboxylic acid; TPT = 2,4,6-tris(4-pyridyl)-1,3,5-triazine; H_3_BTC = trimesic acid (benzene-1,3,5-tricaroxylic acid); H_4_tptc = terphenyl-3,3′′,5,5′′-tetracarboxylic acid; apy = aminopyridine; H_2_peb = 4,4′-[1,4-phenylenebis(ethyne-2,1-diyl)]dibenzoic acid.

The adsorption of iodine in porous materials can be affected by a complex combination of many factors. In general vapour diffusion of iodine into sorbents with high surface areas and large pore volumes exhibit iodine capacities that generally increase in a linear manner with respect to the porosity of the material (Fig. 10). Covalent organic frameworks (COFs) and porous organic polymers (POPs) can exhibit higher iodine capacities compared with MOFs and aerogels with similar (or even higher) porosity (Table 5). Key factors for high iodine adsorption also include the presence of electron-rich surfaces that play a critical role in surface adsorption and the efficient packing of adsorbed iodine molecules within the pores. Thus, frameworks incorporating infinite 1D channels can absorb iodine very effectively owing to more efficient packing of extended iodine-chains within the pores.

**Table 5:** Performance of state-of-the-art porous adsorbents for iodine uptake

Category	Name	BET surface area (m^2^ g^−1^)	Pore volume (cm^3^ g^−1^)	Iodine uptake (g g^−1^)	Ref.
Activated carbon	Activated Carbon	—	—	4.35	157
	Uassis-PC800	3053	1.67	2.25	158
	KOH-AC	1973	1.15	3.76	55
	AC	1292	0.74	2.42	55
	AC	820	0.5	0.76	159
Aerogels	Cg-5C	1200	2.30	2.39	35
	Cg-5P	957	1.62	0.87	35
	MoS_*x*_	370	0.93	1.00	6
	NiMoS	490	1.39	2.25	37
	CoMoS	360	0.50	2.00	37
	SbSnS	240	1.16	2.00	37
	ZnSnS	400	0.77	2.25	37
	KCoS	350	1.17	1.60	37
Porous organic polymers	PAF-23	82	0.04	2.71	52
	PAF-24	136	0.10	2.76	52
	PAF-25	262	0.20	2.60	52
	AzoPPN	400	0.68	2.90	136
	PSIF-5	574	1.41	4.85	137
	SCMP-II	120	0.62	3.45	56
	TTPB	222	0.13	4.43	7
	TTPPA	512	0.30	4.90	51
	TatPOP-2	36.5	0.18	4.50	116
Zeolites	HISL	—	—	0.53	31
	Ag@4A	23.62	0.077	0.160	160
	Bi_5_@Mordenite	412	0.27	0.538	161
	Mordenite	305	0.19	0.275	161
	ZIF-67@MCF	1148	0.76	1.78	162
Covalent organic frameworks	HcOF^a^	—	—	2.90	77
	TPB-DMTP	1927	1.28	6.26	41
	TTA-TTB	1733	1.01	4.95	41
	COF-DL229	1762	0.64	4.7	138
	TPT-DHBD	109	0.30	5.43	42
	SIOC-COF-7	618	0.41	4.81	8
	COF-LZU1	858	0.46	5.30	163
	TpPa1	765	0.48	2.45	163
	Micro-COF-1	816	0.59	2.9	45
	Micro-COF-2	1056	0.71	3.5	45
	Meso-COF-3	982	0.84	4.0	45
	Meso-COF-4	926	1.01	3.3	45

a Studies were conducted for a single crystal and no BET surface area or pore volume was reported.

Recent research has explored methods of overcoming the challenges posed by the practical application of MOFs for the capture of radionuclides. A major concern when MOFs are discussed for industrial applications is that the reactions used to synthesis them require high temperatures, high pressures, long synthesis times and hazardous solvents. These problems have been alleviated using synthesis techniques such as electrosynthesis^113^ and microwave synthesis,^114^ and these methods can be scaled up to make MOFs quickly using continuous flow processes. The use of green solvents has also shown promise in removing the need for hazardous solvents.^115^ Because MOFs are typically produced as powders this makes them impractical for use in industrial scale processes due to problems with handling, contamination and transport. To overcome these problems MOF-polymer composite beads have been fabricated.^116–118^ The beads can be readily handled and have also been shown to increase the iodine capture performance in the case HKUST-1 and PES composites when compared to bulk MOF powder.^116^ Investigations into the stability of MOFs as well as the ability to adsorb and retain iodine under conditions expected during reprocessing and nuclear accidents have also been investigated with promising results. A recent paper confirms that UiO-66-NH_2_ is able to retain iodine under high radiation, temperature and humidity conditions and that the structure was unaffected.^119^ The stability of MOFs when exposed to high levels of radiation^120–122^ has highlighted the potential for them to be used, not only in the capture of radioactive iodine, but also for long-term storage.

MOFs can be considered as promising sorbents for a wide range of small molecules. In this review, we have discussed the emerging understanding of iodine adsorption in MOFs gained from investigations of adsorption methods, MOF design and host–guest chemistry of iodine-loaded systems. We also demonstrate useful strategies for enhancing the sequestration of iodine *via* materials engineering, *e.g.*, glass sintering. Recent research confirms the potential of utilising MOFs in the field of adsorptive capture of radioactive iodine from nuclear fission waste products. However, this area remains largely unexplored due to challenges around the reactive nature of iodine and requirements for MOF stability and difficulties in characterisation of the host–guest systems.

Looking towards the future, the emerging properties of iodine-loaded MOFs also hold great promise for additional practical applications, for example, in catalysis and sensing. Of note is the recent report of the use of MOFs has a carrier of iodine for medical and microbial applications.^164^ ZIF-8 has been immobilised on titanium and can actas a carrier and release agent of iodine for antibacterial therapy in orthopaedics, the release of iodine being triggered by near ir radiation.^165^ Likewise, the photochemical release of dichromate by iodine sorption in a water stable system^166^ reflects the huge potential that MOFs have in developing new polyfunctional and targeted technologies.

## Conflicts of interest

There are no conflicts to declare.

## Supplementary Material
